# Starch characteristics distinguish malt from non-malt barley and can be modulated via *SBEIIb* promoter editing

**DOI:** 10.3389/fpls.2026.1958138

**Published:** 2026-09-30

**Authors:** Mengdi Li, Peixin He, Kangfeng Cai, Jie Yao, Danyin Huang, Guoping Zhang, Lingzhen Ye

**Affiliations:** 1 Zhongyuan Institute, Zhejiang University, Zhengzhou, China; 2 Department of Agronomy, College of Agriculture and Biotechnology, Zhejiang University, Hangzhou, China; 3 Institute of Crop and Nuclear Technology Utilization, Zhejiang Academy of Agricultural Sciences, Hangzhou, China

**Keywords:** amylose/amylopectin ratio, barley (*Hordeum vulgare* L.), CRISPR/Cas9, malt quality, starch

## Abstract

Barley is classified as malt or non-malt barley according to its end use, yet the role of starch characteristics in determining malt quality remains poorly understood. In this study, we systematically compared starch content, composition, granule structure, and thermal properties between six malt and six non-malt barley varieties. Compared with non-malt barley, malt barley exhibited significantly higher total starch (TS) content, a lower amylose/amylopectin (Am/Ap) ratio, and a larger area of large starch granules (LSGs), features that may collectively enhance the contact surface for hydrolytic enzymes during mashing. Thermal property analysis further revealed a narrower gelatinization temperature range (R) in malt barley, indicating more synchronized and potentially more efficient starch gelatinization. Based on these distinct starch traits, we employed novel CRISPR/Cas9-mediated editing of the *SBEIIb* promoter to generate novel barley accessions. The resulting promoter-editing *SBEIIb* mutants showed a significantly reduced Am/Ap ratio, closely mimicking the altered starch profile potentially relevant to malt quality related traits of elite malt barley varieties, without affecting total starch content or grain morphology. Mechanistically, this compositional shift was associated with altered starch-associated genes expression, demonstrating that targeted promoter modification is a promising strategy for malt barley improvement while avoiding the pleiotropic defects commonly caused by coding-region knockouts. These findings reveal fundamental differences in starch properties between malt and non-malt barley and provide both genetic resources and a feasible breeding strategy for developing superior malt barley cultivars.

## 1 Introduction

Barley (*Hordeum vulgare* L.) is the fourth most important cereal crop worldwide, only after wheat, rice, and maize (Yangcheng et al., 2016). It is used mainly as livestock feed and as a raw material for malting and brewing (Chappell et al., 2017). Varieties with superior malting quality are referred to as malt barley, whereas those unsuitable for malt production are termed non-malt barley (Zhao et al., 2022). Due to the stringent quality and flavor requirements of the malting and brewing industries, only about 20% of global barley varieties qualify as malt barley (Nice et al., 2019). Malt barley is generally characterized by rapid and uniform germination, low protein and β-glucan contents, and high hydrolytic enzyme activities, traits closely associated with key malt quality parameters such as diastatic power (DP) and malt extract (Rani and Bhardwaj, 2021). However, these traits do not fully explain the physicochemical basis that distinguishes malt and non-malt barley. Identifying additional grain quality traits that contribute to malting performance is therefore important for improving malt barley in breeding.

Beer production involves malting, mashing, boiling, and fermentation (Yu et al., 2018a, b). During mashing, malt is mixed with hot water to degrade proteins, non-starch polysaccharides, and starch into soluble nutrients and fermentable sugars (Fox, 2016). Barley grains typically contain 50%–72% starch, 2%–10% β-glucan, and 7%–25% protein (Geng et al., 2021, 2022; Li et al., 2021a). Importantly, the breakdown of the malt cell wall, mainly composed of β-glucan and arabinoxylan, is required for amylolytic enzymes to reach the endosperm and initiate starch hydrolysis (Andriotis et al., 2016; Hu et al., 2014). β-Glucan is generally undesirable in malt barley, as high levels increase wort viscosity, reduce filtration rate and extract yield, and promote haze formation in beer (Rani and Bhardwaj, 2021). Efficient degradation of β-glucan during malting is therefore a key characteristic of superior malt barley, resulting in low residual β-glucan content in malt (Wang et al., 2004). Previous studies reported that approximately 80% of grain β-glucan can be degraded during malting and that malt β-glucan content is primarily determined by β-glucanase activity rather than by initial grain content (Kumar et al., 2022; Wang et al., 2004). Proteins can hinder starch gelatinization and hydrolysis by reducing amylase accessibility and entrapping starch granules (Holopainen et al., 2005; Yu et al., 2018b. Consequently, malt quality studies and breeding have focused largely on protein content, β-glucan content, and hydrolytic enzyme activities. In contrast, starch itself, despite being the primary substrate for fermentable sugar production, has received comparatively less attention as a determinant of malt quality.

Starch determines the content and composition of fermentable sugars in wort, directly affecting fermentation efficiency and final product quality (Espinosa-Ramírez et al., 2014; Fox et al., 2019). During mashing, starch gelatinization is a prerequisite for efficient enzymatic hydrolysis, because gelatinized starch is degraded more rapidly and completely than ungelatinized counterpart (Yu et al., 2020). Gelatinization behavior and hydrolysis efficiency are strongly influenced by starch molecular composition, granule size distribution, and thermal properties (Langenaeken et al., 2019; Yu et al., 2018a). Thus, starch characteristics may represent an important but underexplored component of malt quality. However, it remains unclear whether malt barley has a distinct starch profile compared with non-malt barley, and which starch traits, if any, are associated with superior malting performance.

In barley endosperm, starch consists mainly of two glucan polymers: linear amylose (Am) and highly branched amylopectin (Ap). Amylopectin undergoes more extensive hydrolysis than amylose in both corn and barley starches (Naguleswaran et al., 2014). Moreover, smaller amylose molecules and shorter amylopectin chains in barley malt tend to lower gelatinization temperature, likely by improving enzyme accessibility (Yu et al., 2020). In addition, Fredriksson et al. (1998) demonstrated that amylose content is negatively correlated with gelatinization temperature, whereas the weight-average degree of polymerization (DPw) of amylopectin is positively correlated with the gelatinization temperature range (R). Thus, both reduced amylose and shorter amylopectin chains contribute to lower and more synchronous gelatinization. Barley starch granules have a bimodal size distribution: large, lenticular A-type starch granules (LSGs; 10–35 μm in diameter), and small, spherical B-type starch granules (SSGs; 2–7 μm in diameter) (De Schepper et al., 2020). During malting, SSGs are preferentially hydrolyzed, whereas LSG mass remains relatively stable (De Schepper et al., 2020). Enzymatic attack on LSGs begins at discrete surface points and penetrates internally to form pinholes, whereas SSGs undergo mainly surface erosion (Yu et al., 2018b). These findings indicate that both starch molecular structure and granule morphology influence hydrolysis. Nevertheless, systematic comparisons of starch composition, granule structure, and thermal properties between malt and non-malt barley varieties remain limited, hindering a clear understanding of how starch traits contribute to malt quality.

Starch biosynthesis involves multiple enzymes (Huang et al., 2021). Starch branching enzymes (SBEs) catalyze the formation of α-1,6-glycosidic branch points in amylopectin, thereby influencing starch composition, chain-length distribution, and physicochemical properties (Ke et al., 2025; Tetlow and Emes, 2014). In cereal crops, including barley, starch branching enzyme IIb (*SBEIIb*) is predominantly expressed in the endosperm and plays a central role in amylopectin biosynthesis (Sun et al., 1998). Altering *SBEIIb* activity modifies amylopectin branching and the amylose/amylopectin (Am/Ap) ratio, leading to substantial changes in starch structure and functionality (Regina et al., 2010). Thus, *SBEIIb* is a promising target for manipulating starch traits relevant to malt quality. However, complete disruption of starch biosynthesis genes induced by coding-sequence knockout, can cause undesirable pleiotropic effects, such as impaired grain development or reduced starch accumulation (Fahy et al., 2022). In contrast, promoter editing can generate quantitative changes in gene expression while retaining gene function, providing a potentially more moderate approach for adjusting associated traits (Tan et al., 2023). Whether fine-tuning *SBEIIb* expression through promoter editing can generate favorable starch characteristics without severe developmental penalties remains to be tested.

To address these knowledge gaps, we compared starch content, composition, granule structure, and thermal properties between six malt barley and six non-malt barley varieties to identify starch traits associated with malt quality. Based on the starch differences, we then investigated whether targeted modification of the *SBEIIb* promoter could recapitulate favorable starch traits characteristic of malt barley. Using CRISPR/Cas9-mediated promoter editing, we generated barley accessions with altered *SBEIIb* expression and evaluated its starch profile. This study aimed to clarify how starch characteristics distinction between malt from non-malt barley and to establish *SBEIIb* promoter editing as a practical strategy for improving starch-related malt quality traits.

## 2 Materials and methods

### 2.1 Plant materials, malting, and growth conditions

Six elite malt barley varieties (Andreia, Copeland, Metcalfe, Synergy, Irina, and RGT Planet, hereafter “Planet”), and six non-malt barley varieties (ZU9, ZU10, Golden Promise (GP), Hua 30, Yang 1, and Yang 3) were used in this study (**Supplementary Table S1**). Grains were micro-malted following Li et al. (2023).

The barley cultivar GP was used as the recipient for genetic transformation. Plants were grown in a growth chamber under a 14-h light/10-h dark photoperiod (300 μmol m^-^² s^-^¹) and a day/night temperature of 22 °C/18 °C. The growth substrate consisted of a 1:1 (v/v) mixture of peat moss and vermiculite. Immature embryos were harvested 15–20 days post-anthesis for transformation.

### 2.2 Determination of starch, amylose, and β-glucan contents

Mature grains of all barley varieties were ground into flour using a grinder (DFT-50, Dade, China) and stored in a desiccator until analysis. Total starch, amylose, and β-glucan contents were determined as described previously (Geng et al., 2021; Li et al., 2021a). Briefly, Total starch was measured using the K-TSTA kit (AOAC 996.11), based on enzymatic hydrolysis of starch to glucose by thermostable α-amylase and amyloglucosidase, followed by GOPOD colorimetric detection at 510 nm. Amylose content was determined using the K-AMYL kit, which employs Concanavalin A to selectively precipitate amylopectin, allowing quantification of amylose in the supernatant after enzymatic hydrolysis. β-Glucan content was quantified using the K-BGLU kit (AOAC 995.16), involving sequential hydrolysis by lichenase and β-glucosidase to release glucose, which was then measured with GOPOD reagent at 510 nm. Three or four biological replicates were performed for each measurement.

### 2.3 Starch extraction and sieving

Purified starch was extracted according to Yang et al. (2022) with minor modifications. Briefly, cleaned barley grains were soaked in distilled water in 50-mL centrifuge tubes at 4 °C for 24 h. The softened grains were homogenized with distilled water using a portable pulverizer (Jiuyang L3-LJ170, China), and the homogenate was transferred into 2-mL centrifuge tubes for starch purification.

The homogenate was first centrifuged at 4000 rpm for 5 min, and the supernatant was discarded. The pellet was resuspended in 1.8 mL of 80% (w/v) CsCl by vortexing and immediately centrifuged at 4000 rpm for 5 min. This supernatant was discarded, and the CsCl wash was repeated. The starch pellet was then washed sequentially with washing buffer containing 55 mM Tris-HCl (pH 6.8), 2.3% (w/v) SDS, 1% (w/v) dithiothreitol, and 10% (v/v) glycerol, followed by acetone. The purified starch was air-dried at room temperature with the tube lid open, sieved through a 200-mesh sieve, and stored in a desiccator until further analysis.

### 2.4 Scanning electron microscopy of starch granules

Starch samples were sputter-coated with a palladium layer (<10 nm) under vacuum at 7.0 Pa. Images were obtained using a field-emission scanning electron microscope (SU-8010, Hitachi, Tokyo, Japan) at an accelerating voltage of 3 kV. Representative fields containing well-distributed LSGs and SSGs were captured at 1000× magnification.

The numbers of LSGs and SSGs were counted from five SEM images at 1000× magnification. Granule dimensions were analyzed using ImageJ software as described by Ye et al. (2019). For each variety, five SEM images were analyzed, with 10 LSGs and 20 SSGs randomly measured per image. The average from each image served as one independent replicate (n = 5 per variety).

### 2.5 Determination of starch thermal properties

Starch gelatinization properties were determined using a differential scanning calorimeter (DSC Q20, TA Instruments, Newcastle, DE, USA) according to (Ying et al., 2022). Onset temperature (To), peak temperature (Tp), conclusion temperature (Tc), and gelatinization enthalpy (ΔH) were calculated using Universal Analysis 2000 software (version 4.4A).

### 2.6 Bioinformatic analysis of *SBEIIb* and sgRNA design

The genomic sequence of *SBEIIb* (gene ID: *HORVU.MOREX.r3.2HG0170370*) was assembled using Geneious software. The 2.0-kb sequence upstream of the start codon was defined as the putative promoter region. *Cis*-acting regulatory elements within this region were predicted using the PlantCARE online tool (http://bioinformatics.psb.ugent.be/webtools/plantcare/html/). One A-box and one CAAT-box were selected as target motifs for promoter editing.

Guide RNAs (gRNAs) targeting these motifs were designed using the TargetDesign website (http://skl.scau.edu.cn/targetdesign/). Potential off-target sites were evaluated *in silico* using the CRISPOR web tool (http://crispor.tefor.net/crispor.py) (Xie et al., 2015). The sgRNA with the lowest predicted off-target risk among the candidate guides designed by TargetDesign was selected for subsequent editing. The resulting sgRNA sequences were cloned into the pUB-Cas9-U6 vector (He et al., 2025), the sequences of the sgRNAs are provided in **Supplementary Table S2**.

### 2.7 RNA extraction and gene expression analysis

Developing grains of wild-type GP and four mutant lines (Motif1 M1, Motif1 M2, Motif2 M1, and Motif2 M2) were collected at 7, 14, 21, and 28 days after flowering (DAF) for gene expression analysis. For each sample, grains were harvested from at least three independent biological replicates, immediately frozen in liquid nitrogen, and stored at −80 °C until processing.

Total RNA was extracted using the Quick RNA Isolation Kit (Huayueyang Biotechnology, Beijing, China) according to the manufacturer’s protocol. First-strand cDNA was synthesized from 1 μg of total RNA using PrimeScript™ RT Master Mix (Perfect Real Time; Takara, RR036A, Japan). Quantitative real-time PCR (qRT-PCR) was performed on a LightCycler 480 system (Roche, Switzerland) using TB Green^®^ Premix Ex Taq™ II (Tli RNaseH Plus; Takara, RR820A, Japan) to determine *SBEIIb* transcript levels. The barley *GAPDH* gene was used as an internal reference for normalization. Relative expression levels were calculated using the 2^-^ΔΔCT method. Three biological replicates were analyzed for each sample. All gene-specific primers are listed in **Supplementary Table S2**.

### 2.8 Chain-length distribution analysis

The chain-length distribution (CLD) of amylopectin was determined by high-performance anion-exchange chromatography with pulsed amperometric detection (HPAEC-PAD). Starch samples were debranched with isoamylase and analyzed using a Thermo ICS5000+ ion chromatography system (Thermo Fisher Scientific, USA). Cassava starch was used as the quality control and reference standard. Linear glucan standards (Sigma-Aldrich) were used to identify peaks by degree of polymerization (DP). Based on DP, amylopectin chains were classified as A (DP 6–12), B1 (DP 13–24), B2 (DP 25–36), and B3 (DP > 36) (Wang et al., 2025).

The total peak area was calculated by summing the areas of peaks corresponding to glucan chains with DP 6–76. The relative abundance of each chain length was expressed as its percentage of the total peak area.

### 2.9 Determination of branching degree

The branching degree (BD) of amylopectin, defined as the proportion of α-1,6-glycosidic linkages relative to total glycosidic linkages, was determined using proton nuclear magnetic resonance (^1^H NMR) spectroscopy. Purified starch samples (5–10 mg) were dissolved in 1 mL of deuterated dimethyl sulfoxide (D6-DMSO) and heated at 80 °C overnight with continuous shaking to ensure complete dissolution. The samples were then centrifuged at 12,000 rpm for 10 min, and the supernatant was transferred to a 5-mm NMR tube for analysis.

^1^H NMR spectra were recorded on a Bruker BioSpin GmbH spectrometer operating at a ^1^H frequency of 500.23 MHz, with 32 scans acquired at room temperature. Chemical shifts were referenced to the residual DMSO signal at δ 2.50 ppm. Data were processed using MestReNova software. Peak areas corresponding to α-1,4-glycosidic linkages (approximately δ 5.11 ppm) and α-1,6-glycosidic linkages (approximately δ 4.75 ppm) were integrated. BD was calculated using the following formula:


$$BD(\%)=\frac{A}{A+B}\times 100$$

where *A* represents the integrated peak area of α-1,6-glycosidic linkages, and *B* represents the integrated peak area of α-1,4-glycosidic linkages.

### 2.10 Statistical analysis

Statistical analyses were performed using GraphPad Prism 9 (GraphPad Software, USA) and IBM SPSS Statistics 25 (IBM Corp., USA). Pairwise comparisons were conducted using unpaired two-tailed Student’s t-tests. Multiple comparisons were evaluated by one-way or two-way analysis of variance (ANOVA), followed by Tukey’s or Dunnett’s *post-hoc* tests. All figures were generated using GraphPad Prism 9.

## 3 Results

### 3.1 β-Glucan degradation capacity distinguishes malt from non-malt barleys

To assess β-glucan degradation capacity, grains and malt β-glucan contents were determined for 12 barley varieties, and the degradation rate during malting was calculated (**Figure 1a**). In grains, malt barley varieties showed significantly lower β-glucan content than non-malt varieties (*p* < 0.05; **Figure 1b**). After malting, this difference became much more pronounced, with malt barley exhibiting markedly lower β-glucan content than non-malt barley varieties (*p* < 0.0001; **Figure 1c**). Furthermore, malt barley varieties degraded 57%–83% of grain β-glucan, whereas non-malt varieties degraded only 34%–44% (**Figures 1a, d**). Thus, the low β-glucan content in malt barley malt was not solely due to its initial grain β-glucan level but mainly to more efficient degradation during malting. Collectively, these results demonstrate that the two groups can be clearly distinguished by their β-glucan degradation capacity during malting, validating the use of this germplasm panel for subsequent comparisons of starch-related traits.

**Figure 1:**
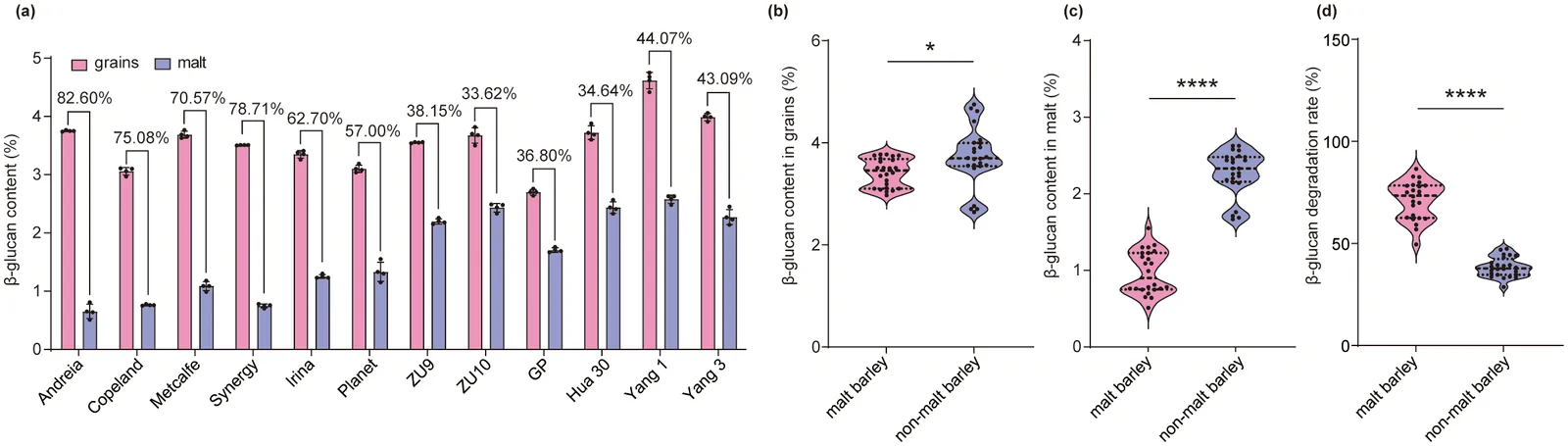
β-Glucan content in grains and malt and its degradation during malting in 12 barley varieties. **(a)** β-Glucan content in barley grains and malt, with values indicating the degradation rate (%) for each variety. **(b)** Grain β-glucan content in malt and non-malt barley varieties. **(c)** Malt β-glucan content in malt and non-malt barley varieties. **(d)** β-Glucan degradation rate during malting in malt and non-malt barley varieties. Malt barley varieties included Andreia, Copeland, Metcalfe, Synergy, Irina, and Planet; non-malt barley varieties included ZU9, ZU10, Yang 3, GP, Hua 30, and Yang 1. Values are presented as means ± SD (n = 4). Asterisks indicate significant differences between malt and non-malt barley varieties: *p<0.05, ****p<0.0001.

### 3.2 Starch content and composition reveal high starch and lower Am/Ap ratio in malt barley

To investigate whether starch-related traits differ between malt and non-malt barley, total starch content and composition were compared among the 12 barley varieties. Malt barley varieties had significantly higher total starch (TS) content (61% - 64%) than non-malt barley varieties (54% - 59%) (**Figures 2a, e**). They also exhibited lower amylose (Am) content (15% - 19%) and correspondingly higher amylopectin (Ap) content (41% - 48%) (**Figures 2b, c, f, g**). Consequently, malt barley had a significantly lower amylose/amylopectin (Am/Ap) ratio than non-malt barley (**Figures 2d, h**). These results indicate that malt barley is characterized not only by higher starch accumulation but also by an amylopectin-enriched starch composition.

**Figure 2:**
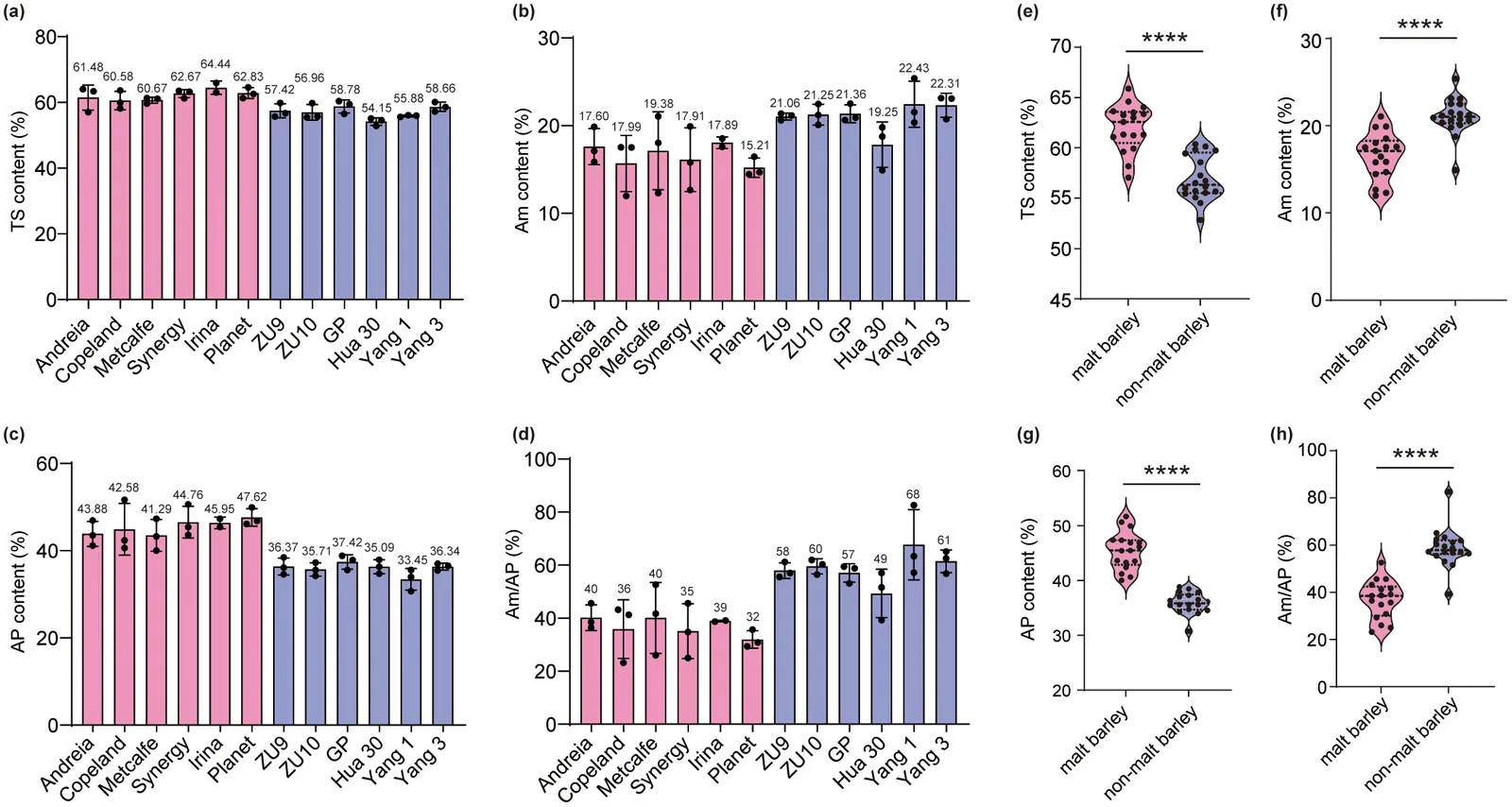
Characterization of starch content and composition in 12 barley varieties. **(a–d)** Total starch (TS), amylose (Am), and amylopectin (Ap) contents (%), and amylose/amylopectin (Am/Ap) (%) ratio in each variety. Values above the bars represent the means of three biological replicates. **(e–h)** Comparisons of TS, Am, and Ap contents and Am/Ap ratio between malt and non-malt barley varieties. Data are presented as means ± SD (n = 3). Asterisks indicate significant differences between malt and non-malt barley varieties: ****p<0.0001 level.

### 3.3 Starch granule size and distribution show larger starch granules but more uniform LSG/SSG proportion in malt barley

To examine starch granule size distribution, purified starch granules from the 12 barley varieties were observed by scanning electron microscopy (SEM), and the proportions and projected areas of SSGs and LSGs were calculated from images acquired at 1000× magnification (**Figure 3**; **Tables 1**, **2**). SSGs were numerically dominant in all barley varieties (**Figures 3a-i**; **Table 1**). Among the six malt barley varieties, LSG proportions ranged narrowly from 9.99% to 13.82%, with SSGs correspondingly at 86.18%–90.01%. In contrast, non-malt barley varieties showed a much broader distribution, with LSG proportions from 7.42% to 22.94% and SSG proportions from 77.06% to 92.58%. Notably, GP and Hua 30 exhibited relatively high LSG proportions, whereas ZU10 and Yang 3 had high SSG proportions (**Table 1**). However, no significant difference was detected between the two groups in the overall proportions of either SSGs or LSGs (**Figures 3j, k**). These results indicate that malt barley varieties display a more uniform granule number distribution, whereas non-malt varieties exhibit greater heterogeneity.

**Figure 3:**
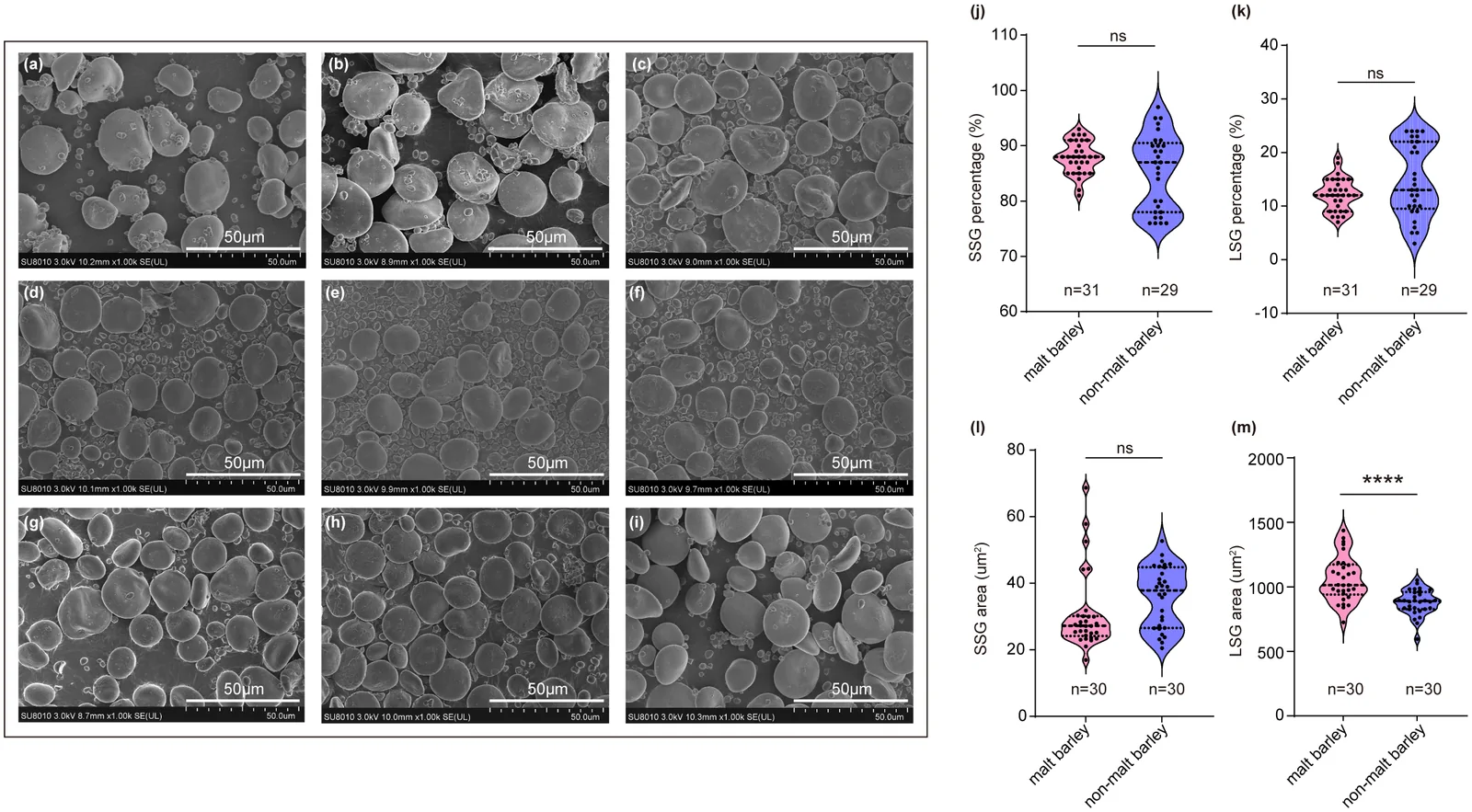
Starch granule profiles of malt and non-malt barley varieties observed by scanning electron microscopy. **(a–i)** Representative SEM images of starch granules from malt barley varieties, including Copeland **(a)**, Synergy **(b)**, and Irina **(c)**, and non-malt barley varieties, including ZU9 **(d)**, ZU10 **(e)**, Yang 3 **(f)**, GP **(g)**, Hua 30 **(h)**, and Yang 1 **(i)**. **(j, k)** Proportions of SSGs and LSGs in SEM images acquired at 1000× magnification. **(l, m)** Projected areas of SSGs and LSGs in SEM images acquired at 1000× magnification, Data are presented as image-level means ± SD (n = 5 images per variety; 6 varieties per group). Malt barley varieties included Andreia, Copeland, Metcalfe, Synergy, Irina, and Planet; non-malt barley varieties included ZU9, ZU10, Yang 3, GP, Hua 30, and Yang 1. Asterisks indicate significant differences between malt and non-malt barley varieties: ****p<0.0001 level; ns, not significant.

**Table 1:** Proportions of small and large starch granules in 1000× SEM fields.

Type	Genotype	LSG	SSG
Malt barley	Andreia	12.92 ± 3.50% bc	87.08 ± 3.50% ab
	Copeland	9.99 ± 1.68% c	90.01 ± 1.68% a
	Metcalfe	13.04 ± 1.47% bc	86.96 ± 1.47% ab
	Synergy	12.82 ± 2.88% bc	87.18 ± 2.88% ab
	Irina	13.82 ± 3.01% bc	86.18 ± 3.01% ab
	Planet	10.11 ± 3.23% c	89.89 ± 3.23% a
Non-malt barley	ZU9	11.55 ± 2.13% bc	88.45 ± 2.13% ab
	ZU10	8.54 ± 1.96% c	91.46 ± 1.96% a
	GP	22.94 ± 0.94% a	77.06 ± 0.94% c
	Hua 30	21.96 ± 1.97% a	78.04 ± 1.97% c
	Yang 1	17.14 ± 5.23% ab	82.86 ± 5.23% bc
	Yang 3	7.42 ± 4.75% c	92.58 ± 4.75% a

Data are presented as means ± SD calculated from five SEM images per variety acquired at 1000× magnification. Different lowercase letters indicate significant differences among varieties within the same column at *p<0.05*.

**Table 2:** Areas of small and large starch granules (μm^2^) in 1000× SEM fields.

Type	Genotype	LSG	SSG
Malt barley	Andreia	971.86 ± 177.37 bc	24.47 ± 4.93 a
	Copeland	1003.39 ± 142.21 b	27.00 ± 2.10 a
	Metcalfe	976.54 ± 79.02 bc	24.30 ± 2.34 a
	Synergy	1286.22 ± 140.19 a	25.80 ± 2.66 a
	Irina	1044.71 ± 95.26 ab	28.72 ± 3.18 a
	Planet	1083.24 ± 223.88 ab	53.53 ± 10.27 d
Non-malt barley	ZU9	867.03 ± 50.96 bc	39.65 ± 4.01 bc
	ZU10	868.87 ± 35.25 bc	42.17 ± 3.31 c
	GP	937.59 ± 73.34 bc	33.60 ± 9.90 ab
	Hua 30	868.38 ± 93.83 bc	28.20 ± 3.09 a
	Yang 1	987.38 ± 25.15 bc	24.74 ± 2.85 a
	Yang 3	748.04 ± 97.73 c	46.92 ± 3.79 c

For each barley variety, granule areas were calculated from five SEM images by measuring 10 LSGs and 20 SSGs per image. Data are presented as means ± SD. Different letters indicate significant differences among varieties at *p<0.05*.

Although the numerical proportions of SSGs and LSGs did not differ significantly between the two groups, granule area analysis revealed a clearer distinction. The projected area of SSGs showed no significant difference between malt and non-malt barley (**Figure 3l**). By contrast, the projected area of LSGs was significantly larger in malt barley than in non-malt barley (**Figure 3m**). Specifically, LSG area ranged from 971.86 to 1286.22 μm² in malt barley and from 748.04 to 987.38 μm² in non-malt barley (**Table 2**). Thus, malt barley was characterized by larger LSGs rather than by a higher proportion of LSGs.

### 3.4 Amylopectin fine structure shows no major differences in chain-length distribution and branching degree between malt and non-malt barley

To determine whether differences in amylopectin molecular architecture contributed to the starch-related variation between malt and non-malt barley, CLD and BD were analyzed. Across the 12 barley varieties, CLD of amylopectin was highly similar (**Supplementary Table S3**). A chains (DP 6–12) accounted for 24.69%–25.00% of total chains, B1 (DP 13–24) for 47.39%–47.60%, B2 (DP 25–36) for 15.53%–15.72%, and B3 (DP > 36) for 11.86%–12.28%. No clear separation between malt and non-malt barley was observed for any chain-length fraction (**Supplementary Table S3**). Similarly, BD varied only slightly among varieties, ranging from 4.89% to 5.80% (**Supplementary Table S3**). Malt barley showed BD values from 4.89% to 5.80%, whereas non-malt barley ranged from 5.06% to 5.66%, indicating that BD did not differ consistently between the two groups.

### 3.5 Thermal properties of starch demonstrate a narrower R in malt barley

Thermal properties of starch from malt and non-malt barley varieties were analyzed to evaluate whether the observed differences in starch composition and granule morphology translated into functional consequences. As shown in **Table 3**, onset temperature (To), peak temperature (Tp), and gelatinization enthalpy (ΔH) ranged from 58.99 °C to 62.49 °C, 65.14 °C to 67.39 °C, and 10.31 to 11.61 J/g, respectively, and did not differ significantly between malt and non-malt barley groups. The similar ΔH values indicate that the overall energy required for gelatinization was broadly comparable between malt and non-malt barley. However, the conclusion temperature (Tc) differed more clearly between the two groups (**Table 3**). Malt barley varieties generally showed lower Tc values than non-malt varieties, resulting in a narrower R (calculated as Tc - To) (**Table 3**). Notably, Andreia, Copeland, Metcalfe, and Irina showed narrow R values of 11.97 °C, 12.11 °C, 13.00 °C, and 13.35 °C, respectively, whereas several non-malt barley varieties (ZU9, Hua 30, Yang 1, and Yang 3) exhibited broader ranges of 17.87 °C, 17.75 °C, 16.84 °C, and 17.91 °C, respectively (**Table 3**).

**Table 3:** Thermal properties of starch determined by differential scanning calorimetry.

Genotype	T_o_ (°C)	T_p_ (°C)	T_c_ (°C)	ΔH (J/g)	PHI	R (°C)
Andreia	61.39 ± 0.07 b	66.58 ± 0.21 bc	73.36 ± 0.06 d	10.53 ± 0.14 a	2.03 ± 0.09 bcd	11.97 ± 0.11 d
Copeland	61.38 ± 0.10 b	66.2 ± 0.13 cde	73.5 ± 0.34 d	10.93 ± 0.45 a	2.27 ± 0.08 a	12.11 ± 0.26 d
Metcalfe	61.31 ± 0.10 b	66.37 ± 0.06 bcd	74.31 ± 0.13 cd	11.24 ± 0.33 a	2.22 ± 0.09 ab	13.00 ± 0.17 d
Synergy	60.81 ± 0.26 b	67.07 ± 0.73 ab	75.88 ± 0.33 b	10.73 ± 0.34 a	1.72 ± 0.08 e	15.07 ± 0.11 c
Irina	59.53 ± 0.19 d	65.56 ± 0.11 ef	72.88 ± 0.33 d	11.12 ± 0.22 a	1.85 ± 0.01 de	13.35 ± 0.36 d
Planet	58.99 ± 0.49 e	66.02 ± 0.61 cde	75.00 ± 1.06 bc	10.31 ± 0.18 a	1.47 ± 0.03 f	16.01 ± 1.14 bc
ZU9	60.2 ± 0.15 c	65.74 ± 0.20 def	78.07 ± 0.99 a	10.96 ± 0.09 a	1.98 ± 0.01 cd	17.87 ± 0.88 a
ZU10	61.14 ± 0.08 b	66.43 ± 0.04 bcd	78.00 ± 0.79 a	10.46 ± 0.53 a	1.98 ± 0.10 cd	16.86 ± 0.86 ab
GP	62.49 ± 0.20 a	67.39 ± 0.23 ab	77.38 ± 0.65 a	10.64 ± 0.22 a	2.17 ± 0.07 abc	14.89 ± 0.78 c
Hua30	59.94 ± 0.15 cd	65.5 ± 0.08 ef	77.69 ± 0.26 a	11.61 ± 0.11 a	2.09 ± 0.04 abc	17.75 ± 0.32 a
Yang1	60.8 ± 0.40 b	65.98 ± 0.30cde	77.65 ± 0.64 a	10.83 ± 0.13 a	2.09 ± 0.03 abc	16.84 ± 0.24 ab
Yang3	59.82 ± 0.42 cd	65.14 ± 0.19 f	77.72 ± 1.29 a	11.15 ± 1.61 a	2.09 ± 0.21 abc	17.91 ± 1.68 a

T_o_: onset temperature; T_p_: peak temperature; T_c_: conclusion temperature; ΔH: enthalpy of gelatinization; PHI (peak height index) = ΔH/(T_p_/T_o_); R = T_c_-T_o_; Data are presented as means ± SD. Different lowercase letters indicate significant differences among varieties within the same column at *p<0.05.*

### 3.6 *SBEIIb* promoter editing generates barley accessions with modified starch profiles

To modify barley starch structure, we performed CRISPR/Cas9-mediated promoter editing of *SBEIIb*, generating mutant lines with alterations in two conserved promoter motifs. Mutations in motif 1 were located upstream of the A-box (containing CCGTCC), whereas those in motif 2 occurred within the CAAT-box (containing CCAAT) (**Figure 4a**). To explore the regulatory significance of these motifs, we performed a promoter analysis of the 2000-bp *SBEIIb* upstream region using PlantPAN 4.0. The predicted *cis*-elements within the two editing target sites revealed that motif 1 is a putative binding site for B3 and/or bZIP transcription factor families, whereas motif 2 is predicted to be recognized by the NF-Y transcription factor family (**Figure 4a**). In addition, the edited grains showed no obvious disruption in grain length or width compared with wild type (WT) (**Figure 4b**), indicating that targeted *SBEIIb* promoter editing did not cause apparent grain morphological effects.

**Figure 4:**
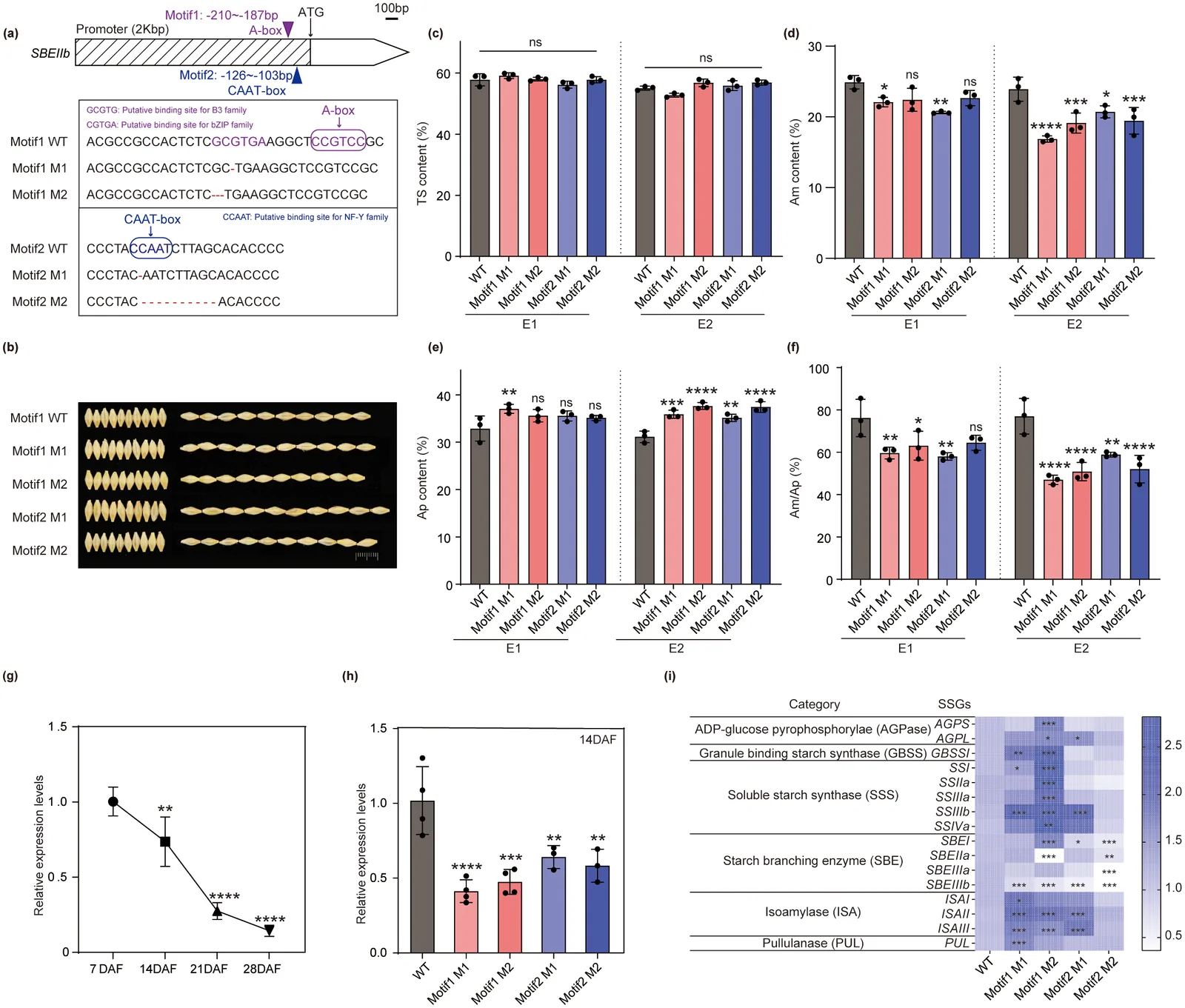
Effects of CRISPR/Cas9-mediated *SBEIIb* promoter editing on starch composition and starch biosynthesis-related gene expression in barley grains. **(a)** Gene structure and schematic representation of the edited sites in two conserved *cis*-regulatory motifs within the *SBEIIb* promoter. Mutations in motif 1 were located upstream of the A-box containing the CCGTCC sequence, whereas mutations in motif 2 occurred within the CAAT-box containing the CCAAT sequence. **(b)** Grain phenotypes of wild-type (WT) and *SBEIIb* promoter-edited mutant lines. Scale bar, 1 cm. **(c–f)** Total starch content (TS), amylose content (Am), amylopectin content (Ap), and amylose/amylopectin ratio (Am/Ap) in WT and mutant grains across two environments, E1 and E2. **(g)** Expression pattern of *SBEIIb* during grain development. **(h)** Relative expression level of *SBEIIb* in WT and mutant grains at 14 DAF. **(i)** Relative expression levels of starch synthesis-related genes in WT and mutant grains at 14 DAF. Data are presented as means ± SD. (n=4). Significance was assessed by one-way ANOVA with Dunnett’s test and two-way ANOVA with Tukey’s multiple-comparisons test. Asterisks indicate significant differences between WT and mutant lines: *p<*0.05*, **p<*0.01*, ***p<*0.001*, and ****p<0.0001; ns, not significant.

To determine whether *SBEIIb* promoter editing altered starch composition, we measured TS and Am contents in WT and mutant grains under two greenhouse environments, and calculated Ap content and the Am/Ap ratio (**Figures 4c–f**). TS content did not differ significantly between WT and the mutants in either environment (**Figure 4c**), suggesting that promoter editing did not markedly affect total starch accumulation. In contrast, Am content was significantly reduced and Ap content increased in the edited lines, resulting in a consistently lower Am/Ap ratio than in WT (**Figures 4d–f**). These compositional changes mirror the starch features of superior malt barley, highlighting the potential of *SBEIIb* promoter editing to shift starch composition toward a malt-favorable profile. We further analyzed *SBEIIb* expression to clarify the regulatory effect of promoter editing. During WT grain development, *SBEIIb* expression progressively declined (**Figure 4g**). At 14 DAF, *SBEIIb* transcript abundance was significantly reduced in the promoter-edited mutants compared with WT (**Figure 4h**). In addition, the expression of several starch metabolism–related genes were also altered in the mutants at 14 DAF, including genes involved in starch biosynthesis and modification such as *GBSSI/Wx*, *SSI*, *SSIIIa*, *SSIIIb*, *SBEI*, *SBEIIa*, *SBEIIIb*, *ISAII*, *ISAIII*, and AGPase-related genes (**Figure 4i**). These results suggest that the changes in starch composition in the promoter-edited mutants were associated not only with reduced *SBEIIb* expression but also with broader transcriptional changes in starch-associated genes.

CLD and BD of amylopectin were also examined in WT and mutant grains. Compared with WT, all promoter-edited mutants showed significantly lower proportions of A and B1 chains and higher proportions of B2 and B3 chains (**Table 4**). BD differed significantly among WT and the promoter-edited mutants (**Table 4**). WT had a BD of 15.90%. Motif1 M1 and Motif1 M2 showed significantly reduced BD values (12.81% and 13.05%, respectively), indicating that editing motif 1 decreased amylopectin branching. In contrast, Motif2 M1 and Motif2 M2 exhibited increased BD values (17.41% and 19.49%, respectively), indicating that editing motif 2 enhanced amylopectin branching, particularly in Motif2 M2. These results indicate that editing different *cis*-regulatory regions of the *SBEIIb* promoter can produce effects on amylopectin branching structure.

**Table 4:** Amylopectin branch chain-length distribution and branching degree in wild-type and *SBEIIb* promoter-edited barley grains.

Genotype	A (DP 6-12)	B1 (DP 13-24)	B2 (DP 25-36)	B3 (DP>36)	BD (%)
WT	27.47 ± 3.08% a	50.55 ± 1.43% a	13.33 ± 0.52% d	8.65 ± 0.17% e	15.9 ± 0.76% b
Motif1 M1	26.66 ± 2.98% d	50.25 ± 1.37% d	13.57 ± 0.52% a	9.52 ± 0.18% b	12.81 ± 1.20% c
Motif1 M2	26.57 ± 2.95% e	50.08 ± 1.35% e	13.48 ± 0.52% b	9.87 ± 0.19% a	13.05 ± 0.84% c
Motif2 M1	27.26 ± 3.05% c	50.50 ± 1.42% b	13.42 ± 0.52% c	8.82 ± 0.18% d	17.41 ± 0.69% b
Motif2 M2	27.38 ± 3.05% b	50.30 ± 1.40% c	13.34 ± 0.52% d	8.98 ± 0.18% c	19.49 ± 0.61% a

We further assessed whether the altered starch composition and amylopectin structure were accompanied by changes in starch granule morphology or thermal properties. No significant differences were observed in the proportions or projected areas of SSGs and LSGs between WT and the mutants, and SEM images showed no apparent morphological changes (**Supplementary Figure S1**). Regarding thermal properties, although some individual parameters differed among genotypes, the R did not differ significantly between WT and mutants (**Supplementary Table S4**). These results suggest that *SBEIIb* promoter editing mainly affected starch composition and amylopectin molecular structure, with limited effects on granule-level morphology and overall R under the tested conditions.

## 4 Discussion

Starch is the most abundant carbohydrate in barley grains, accounting for approximately 50%–72% of grain dry weight (Li et al., 2021). As the principal substrate for fermentable sugar production during mashing, starch content and composition may influence traits relevant to malting performance (Fox and Bettenhausen, 2023). In the present study, the selected malt barley varieties had higher TS content, lower Am content, higher Ap content, and a lower Am/Ap ratio than the selected non-malt barley varieties. These differences identify a starch compositional pattern associated with the malt barley materials evaluated here.

Higher total starch content provides a larger substrate pool for amylolytic enzymes, potentially increasing fermentable sugar production (Hu et al., 2021). In contrast, amylose content is generally negatively associated with starch solubilization and hydrolysis efficiency; high-amylose starches often exhibit restricted gelatinization and reduced enzymatic digestibility, leading to lower fermentable sugar yields (Yu et al., 2018b). Therefore, the combination of higher TS content, lower Am content, and a reduced Am/Ap ratio may promote more efficient starch conversion during mashing. These compositional features represent important traits that distinguish malt barley from non-malt barley and may partly explain the superior brewing suitability of malt barley.

The relative abundance and morphology of SSGs and LSGs influence starch gelatinization and enzymatic hydrolysis, thereby affecting susceptibility to enzymatic hydrolysis during mashing and ultimately malting and mashing performance (De Schepper and Courtin, 2022; Langenaeken et al., 2019). SSGs are generally considered more readily hydrolyzed than LSGs due to their larger surface-to-volume ratio, which enhances contact with amylolytic enzymes and facilitates more complete hydrolysis (De Schepper et al., 2020; Tang et al., 2004; Yu et al., 2020). Previous studies have reported that SSGs account for 95%–98% of starch granules in several commercial malt barley varieties (De Schepper et al., 2020). However, in the present study, several non-malt barley varieties in the present study showed SSG proportions within or even above the range observed in malt barley varieties, yet they did not display correspondingly superior malt-related characteristics. This suggests that the proportion of SSGs is not a key trait for determining malt quality. One possible explanation is that SSGs may become entrapped within the protein matrix, limiting enzyme accessibility and resulting in incomplete hydrolysis even under favorable gelatinization conditions (Langenaeken et al., 2019). Therefore, starch hydrolysis efficiency during mashing is likely determined by multiple interacting factors, including granule morphology, starch molecular structure, protein distribution, β-glucan degradation, and amylolytic enzyme accessibility. Previous study has demonstrated that large starch granules yield relatively more fermentable sugars during mashing (Langenaeken et al., 2019). Therefore, the relatively uniform granule distribution and larger LSG area observed in malt barley may represent structural features associated with more consistent and efficient starch utilization during brewing.

Amylopectin fine structure is a key determinant of starch physicochemical properties, including gelatinization behavior (Li and Gong, 2020), crystalline stability (Li and Gong, 2021), and enzymatic digestibility (Srichuwong et al., 2005). Its CLD of amylopectin influences the formation and stability of double helices within crystalline lamellae, while the BD reflects the proportion of α-1,6-glycosidic linkages (Jane et al., 1999; Vamadevan and Bertoft, 2015; Yang et al., 2017). However, our results suggest that the major starch differences between malt and non-malt barley are not primarily due to large alterations in amylopectin CLD or BD (**Supplementary Table S3**). Instead, the distinguishing starch features identified here are more closely associated with TS content, Am/Ap ratio, and granule morphology. Nevertheless, because amylopectin fine structure is known to influence gelatinization and enzymatic hydrolysis, even subtle differences in chain organization, crystalline packing, or interactions with amylose and non-starch components may still contribute to starch functionality during mashing (Jane et al., 1999; Tester and Morrison, 1990; Vamadevan and Bertoft, 2015).

Gelatinization is a prerequisite for efficient starch hydrolysis during mashing because native starch granules must swell and lose crystalline order before being effectively degraded by amylolytic enzymes (De Schepper et al., 2020; Langenaeken et al., 2019). In this study, the combination of a narrower R and similar ΔH suggests that starch in malt barley undergoes more rapid and synchronized gelatinizations (**Table 3**). A narrower R value reflects greater uniformity in granular structure or crystallite stability, causing granules to complete gelatinization within a smaller temperature interval (Langenaeken et al., 2019). This property may be advantageous during mashing, as synchronized gelatinization facilitates timely granule swelling, disruption of crystalline regions, and subsequent enzymatic hydrolysis. By contrast, the broader R and higher Tc values in non-malt barley suggest delayed gelatinization completion, which may restrict enzyme accessibility and reduce starch conversion efficiency.

The differences in thermal properties observed between malt and non-malt barley are likely caused by multiple starch structural characteristics. Previous studies have shown that gelatinization behavior is influenced by amylose content, amylopectin fine structure, granule size, crystalline organization, lipid–amylose complexes, and interactions with proteins and non-starch polysaccharides (Jane et al., 1999; Tester and Morrison, 1990; Vamadevan and Bertoft, 2015; Yu et al., 2018a). High-amylose cereals often exhibit altered gelatinization parameters (Li et al., 2021b), and *SSIIa* mutants with increased amylose content show decreased To, Tp, Tc, and ΔH values (Yang et al., 2022). In the present study, non-malt barley had higher amylose content than malt barley, yet To, Tp, and ΔH did not differ significantly between the two groups, whereas Tc was elevated in non-malt barley. These findings highlight the complexity of starch gelatinization, which cannot be explained by amylose content alone. The amylopectin fine-structure results further support this interpretation. Because amylopectin CLD and BD were highly similar between malt and non-malt barley (**Supplementary Table S3**), the distinct gelatinization behavior is unlikely to be driven primarily by differences in amylopectin molecular architecture. Instead, the broader R and delayed completion in non-malt barley may be partially attributable to the combined effects of a higher Am/Ap ratio and smaller LSG area. These starch characteristics, together with possible differences in crystalline organization and starch–matrix interactions, may reduce gelatinization uniformity and limit efficient hydrolysis during mashing. Collectively, malt barley starch profiles are characterized by higher total starch content, lower Am/Ap ratio, larger LSG area, and narrower R. These features may be relevant to starch gelatinization and enzymatic hydrolysis during mashing. However, whether these characteristics result in increased fermentable sugar production or improved wort quality remains to be experimentally determined.

Promoter editing of *SBEIIb* provided an opportunity to examine whether starch-related traits could be modified without apparent effects on total starch accumulation or grain morphology. The promoter-edited lines maintained total starch content and starch-granule morphology comparable to those of WT, while showing lower amylose content, higher amylopectin content, and a reduced Am/Ap ratio (**Figures 4c–f**; **Supplementary Figure S1e**). One possible explanation is that promoter editing quantitatively altered *SBEIIb* expression rather than completely eliminating *SBEIIb* function. Such partial modulation may affect amylopectin biosynthesis and starch composition while retaining sufficient branching activity to sustain overall starch accumulation. In addition, starch biosynthesis is regulated by a coordinated network of branching enzymes, starch synthases, debranching enzymes, and associated regulatory factors. Functional compensation or coordinated regulation within this network may have buffered the effects of altered *SBEIIb* expression on grain morphology and total starch content (Regina et al., 2010). These explanations are biologically plausible but require further experimental validation.

Consistent with the altered starch composition, all promoter-edited mutants showed significantly lower proportions of A and B1 chains and higher proportions of B2 and B3 chains with WT. Previous studies suggested that shorter amylopectin chains form weaker or shorter double helices, leading to less stable crystalline structures, shorter gelatinization times, and enhanced enzymatic hydrolysis, whereas longer chains may increase gelatinization time and reduce enzymatic hydrolysis efficiency (Zhu, 2018). Therefore, the higher proportion of longer amylopectin chains in the edited lines should not be interpreted as direct evidence of altered mashing efficiency without further functional analysis.

BD also differed significantly among WT and the promoter-edited mutants (**Table 4**). The contrasting branching phenotypes between the two motif classes may be associated with differences in the regulatory contexts of the edited promoter regions. *In silico* analysis using PlantPAN 4.0 suggested that motif 1 may contain potential binding sites for B3 and/or bZIP transcription factors, whereas motif 2 may be associated with NF-Y transcription factors. These distinct predicted regulatory features could potentially influence *SBEIIb* expression and, consequently, amylopectin branching and starch structure. However, direct transcription-factor binding, promoter activity, and the underlying molecular mechanisms have not been experimentally validated. In addition, compensatory changes in other starch-biosynthetic enzymes may also have contributed to the observed phenotypes.

Taken together, targeted editing of *cis*-regulatory elements within the *SBEIIb* promoter reduced Am content, increased Ap content, lowered the Am/Ap ratio, and altered amylopectin CLD and BD without causing apparent defects in grain morphology or total starch accumulation. These findings demonstrate that *SBEIIb* promoter editing can modulate starch composition and structure, thereby providing a potential genetic strategy for modifying starch-related traits relevant to malt quality. However, the promoter-edited lines used in this study are currently transgenic materials and therefore could not be cultivated under natural field conditions to produce sufficient grains for malting and comprehensive malt quality evaluation. As a result, the direct effects of the altered starch traits on malting performance and brewing quality remain to be determined. Future work should focus on generating non-transgenic edited lines, increasing seed availability, and evaluating their malting and mashing performance under field-relevant conditions.

Several limitations should also be considered when interpreting the comparative analysis of malt and non-malt barley. The varieties analyzed in this study differed in genetic background; therefore, the observed starch differences may reflect both malting classification and background-dependent genetic variation. The present findings should consequently be interpreted as associations identified within the selected germplasm rather than universal characteristics of all malt and non-malt barley. Validation using a larger and genetically diverse panel, or genetically matched materials, together with direct correlation analyses between starch traits and standard malting-quality parameters, will be required to establish the broader predictive value of these starch characteristics.

## Data Availability

The datasets presented in this study can be found in online repositories. The names of the repository/repositories and accession number(s) can be found in the article/**Supplementary Material**.

## Supplementary material

Supplementary Figure 1 Starch granule morphology and size distribution in wild-type and *SBEIIb* promoter-edited barley grains. **(a, b)** Proportions of SSGs and LSGs in SEM images acquired at 1000× magnification. **(c, d)** Projected areas of SSGs and LSGs in SEM images acquired at 1000× magnification. **(e)** Representative SEM images of starch granules from WT and *SBEIIb* promoter-edited mutant lines. Data are presented as means ± SD. Asterisks indicate significant differences between WT and mutant lines: * *p<0.05*; ns, not significant.
