# Supplementary material for: Starch characteristics distinguish malt from non-malt barley and can be modulated via SBEIIb promoter editing
Source: Front Plant Sci. 2026 Sep 30;17:1958138. doi: 10.3389/fpls.2026.1958138 (PMC13662047; doi:10.3389/fpls.2026.1958138)
Supplement: Supplementary file 1 [file Table1.docx]

| Supplementary table 1 Information of 12 barley cultivars. | | | | | | |
| --- | --- | --- | --- | --- | --- | --- |
| Genotype | Origin | End-use | Row-type | Variety Characteristics at Registration | Breeding Unit | Reference |
| Andreia | Argentina | Malting | 2-row | Diastatic power: 254W.K.; Malt extraction rate: 80.7%; α-Amino nitrogen: 187mg/100g; β-Glucan: 90mg/L. | unknown | 陆东华,何加兵,韦宁.阿根廷大麦麦芽Andreia的应用研究[J].中外酒业,2025,(11):20-23. |
| CDC Copeland (Copeland) | Canada | Malting | 2-row | Protein: 11.6% Diastatic power: 157°; Malt extraction rate: 80.8%; Free Amino Nitrogen: 187mg/L; β-Glucan: 91mg/L; Viscosity: 1.44cP; Kolbach Index: 39.8%. | Crop Development Centre at the University of Saskatchewan | <https://grainscanada.gc.ca/en/grain-research/export-quality/cereals/malting-barley/2021/quality-data.html> |
| AC Metcalfe (Metcalfe) | Canada | Malting | 2-row | Protein: 12.1% Diastatic power: 188°; Malt extraction rate: 81.1%; Free Amino Nitrogen: 202mg/L; β-Glucan: 93mg/L; Viscosity: 1.43cP; Kolbach Index: 39.9%. | Agriculture and Agri-Food Canada | <https://grainscanada.gc.ca/en/grain-research/export-quality/cereals/malting-barley/2021/quality-data.html> |
| AAC Synergy (Synergy) | Canada | Malting | 2-row | Protein: 11.3% Diastatic power: 161°; Malt extraction rate: 81.3%; Free Amino Nitrogen: 176mg/L; β-Glucan: 69mg/L; Viscosity: 1.42cP; Kolbach Index: 40.2%. | Agriculture & Agri-Food Canada | <https://grainscanada.gc.ca/en/grain-research/export-quality/cereals/malting-barley/2021/quality-data.html> |
| Irina | Russia | Malting, Feeding | 2-row | Protein: 15.30% Diastatic power: 400.85W.K.; Malt extraction rate: 77.13%; α-Amino nitrogen: 172.40mg/100g; β-Glucan: 258.60mg/L; Kolbach Index: 33.41%. | KWS SAAT SE & Co. KGaA (KWS) | Bao et al., 2024, Journal of Plant Genetic Resources, DOI：10.13430/j.cnki.jpgr.20240202004 |
| RGT Planet (Planet)) | France | Malting | 2-row | Protein: 11.34% Diastatic power: 293.37W.K.; Malt extraction rate: 82.04%; α-Amino nitrogen: 138.64mg/100g; β-Glucan: 483.11mg/L; Kolbach Index: 41.09%. | French company RAGT Semences (RAGT), Seed Force | Bao et al., 2024, Journal of Plant Genetic Resources, DOI：10.13430/j.cnki.jpgr.20240202004 |
| Genotype | Origin | End-use | Row-type | Variety Characteristics at Registration | Breeding Unit | Reference |
| Zheda NO.9 (ZU9) | China | Feeding, Malting | 2-row | Crude protein: 10.3%; Malt extraction rate: 80.0%; Kolbach index: 46.2%; Other quality traits: unknown. | Zhejiang University | <http://www.xnysyz.zju.edu.cn/2023/0423/c81133a2904389/page.htm> |
| Zheda NO.10 (ZU10) | China | Feeding | 2-row | Average yields: 257.6 and 311.2 kilograms/mu;  Other quality traits: unknown. | Zhejiang University | <http://www.xnysyz.zju.edu.cn/2022/0624/c81133a2904387/page.htm> |
| Golden Promise (GP) | Britain | Malting | 2-row | Protein: 13.8% Diastatic power: 504.31W.K.; Malt extraction rate: 75.08%; α-Amino nitrogen: 143.17mg/100g; β-Glucan: 274.63mg/L; Kolbach Index: 31.37%. | Miln Marsters seed company of Cheshire | Bao et al., 2024, Journal of Plant Genetic Resources, DOI：10.13430/j.cnki.jpgr.20240202004 |
| Hua30 | China | Feeding | 2-row | Crude protein: 13.6%; Crude fiber: 5.3%; Crude ash: 2.3%;  Other quality traits: unknown. | The Institute of Biotechnology of Shanghai Academy of Agricultural Sciences and the Agricultural Science Research Institute of Jiaxing City, Zhejiang Province | <http://shuju.aweb.com.cn/breed/20121011/180614.html> |
| Yangsimai NO.1 (Yang1) | China | Feeding | 2-row | Thousand-grain weight: 30g; Protein: 13.9%;  Other quality traits: unknown. | Yangzhou University | <https://www.sme-gov.cn/yangzhou-chengguo-59090.html> |
| Yangsimai NO.3 (Yang3) | China | Feeding | 2-row | Protein: 15.56%; Starch:58.84% Lysine: 0.58%; β-Glucan: 5.84%;  Other quality traits: unknown. | Yangzhou University | <https://news.ymt.com/arg_tech/Nmlz1L8e> |

| Supplementary table 2 Primers list for gene editing and expression levels analysis. | | | | | | | |  |
| --- | --- | --- | --- | --- | --- | --- | --- | --- |
| Primer Name | | Sequence | | | | Application | |  |
| Motif1-F | | cttgCGCCGCCACTCTCGCGTGA | | | | CRISPR/Cas9 | |  |
| Motif1-R | | aaacTCACGCGAGAGTGGCGGCG | | | |  |  |  |
| Motif2-F | | cttgGGGTGTGCTAAGATTGGTA | | | |  |  |  |
| Motif2-R | | aaacTACCAATCTTAGCACACCC | | | |  |  |  |
| *GAPDH*-F | | GTGAGGCTGGTGCTGATTACG | | | | RT-qPCR | |  |
| *GAPDH*-R | | TGGTGCAGCTAGCATTTGAGAC | | | |  |  |  |
| *AGPS*-RTF | | TTGTGGCTATTCGAGGAG | | | |  |  |  |
| *AGPS*-RTR | | CATCCGTTTCTCTGTGTG | | | |  |  |  |
| *AGPL*-RTF | | GTAATGCTCGGTGCTGAT | | | |  |  |  |
| *AGPL*-RTR | | TGAATCGAAGTGTTCTCCC | | | |  |  |  |
| *GBSSI*-RTF | | GCATCCACAACATCTCGTA | | | |  |  |  |
| *GBSSI*-RTR | | TCGTAGCCGTCAATGAAG | | | |  |  |  |
| *SSI*-RTF | | CTACTTTGCTATGCTGCTTG | | | |  |  |  |
| *SSI*-RTR | | CATGCCAATCGTTCACAAC | | | |  |  |  |
| *SSIIa*-RTF | | AAATGATTGGCACACGGCAC | | | |  |  |  |
| *SSIIa*-RTR | | GCCCTGGTGAGCGATGTTAT | | | |  |  |  |
| *SSIIIa*-RTF | | ATCTATGACAACAGGAATGG | | | |  |  |  |
| *SSIIIa*-RTR | | CGACGGCAATATGGATAA | | | |  |  |  |
| *SSIIIb*-RTF | | GATGTGGACGATGATAAGG | | | |  |  |  |
| *SSIIIb*-RTR | | GAGCATAATCTACGCCATT | | | |  |  |  |
| *SSIVa*-RTF | | CAAGGTCCAGTATAATGCTAAG | | | |  |  |  |
| *SSIVa*-RTR | | GTGGTTGTGAAGGATATGC | | | |  |  |  |
| *SBEI*-RTF | | TTGGCGACAAGACTATGG | | | |  |  |  |
| *SBEI*-RTR | | ATTGTAGGTGAAGCAGGC | | | |  |  |  |
| *SBEIIa*-RTF | | TTTAGGGATGAGGTGCTG | | | |  |  |  |
| *SBEIIa*-RTR | | GTATGAATGCTCCTGGATTG | | | |  |  |  |
| *SBEIIb*-RTF | | GAACACCGCCTTCCATCG | | | |  |  |  |
| *SBEIIb*-RTR | | CCGAATAGTATCTTCACCTCCAT | | | |  |  |  |
| *SBEIIIa*-RTF | | TGTCCATAGCCATGCAAGCA | | | |  |  |  |
| *SBEIIIa*-RTR | | CTTGTGCGCTTTGTCCAACA | | | |  |  |  |
| *SBEIIIb* -RTF | | CCAGAAGCAACTGTTGTGGC | | | |  |  |  |
| *SBEIIIb* -RTR | | TCCACCTTCATCAACTGGCC | | | |  |  |  |
| *ISAI*-RTF | | GAAACCTTGGCACAGTATC | | | |  |  |  |
| *ISAI*-RTR | | TCCATCTCTGTTGTCCTC | | | |  |  |  |
| *ISAII*-RTF | | GTCAAGGAAGTTACTGGATT | | | |  |  |  |
| *ISAII*-RTR | | CGTGGTTCAGATAGATTGT | | | |  |  |  |
| *ISAIII*-RTF | | TTGGCAACTCGTATATCTG | | | |  |  |  |
| *SAIII*-RTR | | GATACAAGGTCACATAAGGT | | | |  |  |  |
| *PUL*-RTF | | CTTATGTCGTAACCGTCTT | | | |  |  |  |
| *PUL*-RTR | | CAGATTTCCTCACCAAAGTA | | | |  |  |  |
| Supplementary table 3 Amylopectin chain-length distribution and branching degree in malt and non-malt barley varieties. | | | | | | | | |
| Genotype | A （DP 6-12） | | B1 （DP 13-24） | B2 （DP 25-36） | B3 （DP>36） | | BD (%) | |
| Andreia | 24.69% | | 47.46% | 15.56% | 12.28% | | 4.89 | |
| Copeland | 24.88% | | 47.51% | 15.55% | 12.06% | | 5.36 | |
| Metcalfe | 24.92% | | 47.53% | 15.59% | 11.97% | | 5.24 | |
| Synergy | 24.93% | | 47.57% | 15.56% | 11.94% | | 5.04 | |
| Irina | 24.96% | | 47.56% | 15.56% | 11.92% | | 5.80 | |
| Planet | 25.00% | | 47.60% | 15.53% | 11.87% | | 5.25 | |
| ZU9 | 25.00% | | 47.56% | 15.56% | 11.88% | | 5.36 | |
| ZU10 | 24.96% | | 47.56% | 15.59% | 11.89% | | 5.28 | |
| GP | 24.96% | | 47.55% | 15.60% | 11.90% | | 5.66 | |
| Hua30 | 24.99% | | 47.59% | 15.56% | 11.86% | | 5.25 | |
| Yang1 | 24.97% | | 47.55% | 15.57% | 11.91% | | 5.06 | |
| Yang3 | 24.98% | | 47.39% | 15.72% | 11.91% | | 5.29 | |

| Supplementary table 4 Thermal properties of starch from wild-type and *SBEIIb* promoter-edited barley grains determined by differential scanning calorimetry (DSC). | | | | | | |
| --- | --- | --- | --- | --- | --- | --- |
| Genotype | To(℃) | Tp(℃) | Tc(℃) | ΔH (J/g) | PHI | R(℃) |
| WT | 60.19±0.03 a | 64.94±0.19 a | 70.48±0.40 a | 9.25±0.15 a | 1.95±0.08 a | 10.29±0.43 a |
| Motif1 M1 | 62.90±0.46 c | 68.43±0.26 b | 74.67±0.70 b | 7.23±0.70 a | 1.30±0.07 b | 11.77±0.73 a |
| Motif1 M2 | 61.97±0.10 bc | 67.27±0.16 ab | 72.58±0.35 ab | 8.82±0.55 a | 1.66±0.03 a | 10.62±0.44 a |
| Motif2 M1 | 61.11±1.41 ab | 66.57±2.08 ab | 73.00±3.22 ab | 11.23±3.55 a | 2.02±0.38 a | 11.90±1.82 a |
| Motif2 M2 | 60.31±0.16 a | 65.51±0.30 a | 71.44±0.52 ab | 9.37±0.32 a | 1.80±0.06 a | 11.13±0.42 a |
| To: onset temperature; Tp: peak temperature; Tc: conclusion temperature; ΔH: enthalpy of gelatinization; PHI (peak height index)=ΔH/(Tp/To);R (gelatinization range)=Tc-To; Data are presented as means ± SD. Different lowercase letters indicate significant differences among varieties within the same column at *p<0.05*. | | | | | | |
